# The Role of Sub- and Supercritical CO_2_ as “Processing Solvent” for the Recycling and Sample Preparation of Lithium Ion Battery Electrolytes

**DOI:** 10.3390/molecules22030403

**Published:** 2017-03-06

**Authors:** Sascha Nowak, Martin Winter

**Affiliations:** 1 MEET Battery Research Center, University of Muenster, Corrensstraße 46, 48149 Münster, Germany; martin.winter@uni-muenster.de; 2 Helmholtz Institute Münster, IEK-12, Forschungszentrum Jülich, Corrensstraße 46, 48149 Münster, Germany

**Keywords:** supercritical CO_2_, subcritical CO_2_, liquid CO_2_, lithium ion battery electrolytes, lithium ion battery, recycling, sample preparation, aging, post-mortem

## Abstract

Quantitative electrolyte extraction from lithium ion batteries (LIB) is of great interest for recycling processes. Following the generally valid EU legal guidelines for the recycling of batteries, 50 wt % of a LIB cell has to be recovered, which cannot be achieved without the electrolyte; hence, the electrolyte represents a target component for the recycling of LIBs. Additionally, fluoride or fluorinated compounds, as inevitably present in LIB electrolytes, can hamper or even damage recycling processes in industry and have to be removed from the solid LIB parts, as well. Finally, extraction is a necessary tool for LIB electrolyte aging analysis as well as for post-mortem investigations in general, because a qualitative overview can already be achieved after a few minutes of extraction for well-aged, apparently “dry” LIB cells, where the electrolyte is deeply penetrated or even gellified in the solid battery materials.

## 1. Introduction

Due to unrivalled high energy densities and high specific energies, lithium ion batteries (LIBs) are used in everyday modern portable consumer electronics, such as tablets, smartphones and laptops. Furthermore, they are considered the most promising battery technology for pure electric and hybrid electric vehicles (xEVs) [1,2,3] and, due to high energy efficiencies, also for stationary energy storage applications [4]. This widespread application for private and industrial purposes will inevitably raise a demand for reutilization and recycling of the lithium ion battery components. Due to the high prices of Ni and Co, which are deployed in the form of oxides in the LIB cathode, and of Cu used as current collector at the anode [5], recycling is also of economic interest. Recycling processes for the metals in the cathode already exist. The recycling value of these metals would, however, be increased if direct recovery as ready to use battery raw constituents were possible. However, the high purity requirements of the battery material manufacturers diminish this approach. In addition, the recycling of LIBs or its single constituents is encouraged by legislation. Lithium, for example, has no actual substitute and due to its growing application, the demands might exceed the global production by the 2020s [6,7]. Therefore, Li recycling needs to be established. For the EU, limited occurrences were reported, e.g., in Portugal [8,9] or the Czech Republic [10]. Thus, the European Parliament and Council of the European Union issued several directives such as the Waste of Electrical and Electronic Equipment (WEEE) 2012/19/EU and the End of Life Vehicles (ELV) 2000/53/EC, which deal with the recycling of batteries from electronic products and electric vehicles. Beginning in 2016, at least 45 wt % of electrical and electronic equipment needs to be collected by the EU members. The reuse and recovery rate for end of life vehicles is set to at least 85% regarding both the weight per vehicle and the calendar year. Furthermore, the Battery Directive 2006/66/EC was instituted as the most advanced battery recycling legislation worldwide. Each EU member state has to meet a collection rate of 45% and at least a recycling efficiency of 50 wt % for non-lead-acid and non-nickel-cadmium batteries [11,12,13].

With regard to upcoming pilot processes and plants, the LIB aging has to be taken into account. New or only lightly used LIBs from electric vehicles are not the focus of recycling strategies. Foremost, damaged batteries or batteries that have achieved the end of life (EOL), i.e., strongly aged batteries, are in the focus of recycling. Aging is one of the main performance deteriorations of LIBs as aging leads to capacity loss, resistance increase, power and energy loss and therefore to a reduced lifetime [14,15]. Aging may also be responsible for safety changes of batteries [16]. However, there is no “universal aging mechanism”; in fact, numerous aging mechanisms occur and they can affect each other. Reports in the literature usually focused on the individual parts of the LIB cell such as the reactions between the electrolyte and the anode, solid electrolyte interphase (SEI) growth, the decomposition of cathode and anode, or lithium metal deposition on the anode [17,18,19,20,21,22,23,24,25,26,27,28,29] or on the interaction between the different materials [30]. Additionally, the operating conditions have a great influence of the degradation behavior [31,32,33]. However, the decomposition of the electrolyte is challenging to investigate due to its complex composition. Because of the sensitivity towards water and thermal influence, the literature reveals numerous reports about aging products and mechanisms. The variety of decomposition products includes HF [34,35,36,37,38,39,40], inorganic and organic (fluoro)phosphates (OPs) [41,42,43,44,45,46,47,48], CO_2_ [49,50], dicarboxylates and oligocarbonate based products [51,52,53], diols [54] and alkyl fluorides [36,45,50]. Furthermore, the applied analysis methods and corresponding reaction mechanisms have been intensively discussed in literature and new reports are constantly added [55,56].

Especially, the fluorinated decomposition compounds and, in particular HF, are in special focus with regard to pilot processes and plants, since, due their chemically aggressive nature and their toxicity, they can seriously hamper or damage the industrial recycling approaches. Therefore, these compounds need to be removed before the recycling process. However, aged LIBs, where the liquid electrolyte is partially decomposed into solid and gaseous products, often appear as “dry”. During operation, the electrolyte immobilizes into the deeper layers of the electrode and into the solid electrolyte decomposition products and can therefore not easily be recovered either for simple removal or subsequent analysis.

Sub- and supercritical CO_2_ are attractive extraction tools for overcoming both challenges. While it is still a young research field, the usage of CO_2_ as a recycling or sample preparation tool for the recovery and analysis of materials is repeatedly found in the literature in the last years.

## 2. Lithium Ion Batteries Electrolytes

**Figure 1:**
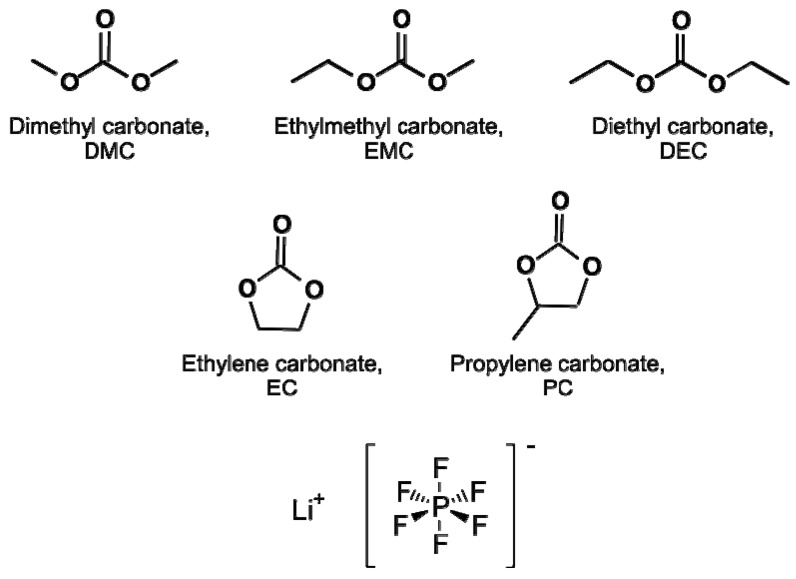
Structures of six of the most important linear and cyclic organic carbonates and the conducting salt lithium hexafluorophosphate for the electrolyte system in lithium ion batteries [60,61].

Lithium ion batteries consist of a carbon/graphite based anode, a lithium transition metal oxide cathode and an electrolyte soaked polyolefin-based separator [57,58]. The electrolyte inside a lithium ion battery has to fulfill several requirements: wide electrochemical stability window, high ionic conductivity and redox stability are some of the desired requirements [59]. Additionally, the chemical and electrochemical compatibility with the other cell constituents should be ensured; furthermore, the electrolyte should be non-toxic, safe, environmentally friendly and cost efficient [60,61]. In order to meet these requirements, the electrolyte system typically consists of a conducting salt (1 M), dissolved in a mixture of different linear carbonates, e.g., dimethyl carbonate (DMC), ethyl methyl carbonate (EMC) or dimethyl carbonate (DEC), and a cyclic carbonate such as propylene carbonate (PC) or ethylene carbonate (EC) [62,63,64] (Figure 1). The most commercially applied conducting is lithium hexafluorophosphate (LiPF_6_) (Figure 1) while alternatives such as lithium tetraborate (LIBF_4_), lithium bis-(oxalato)borate (LiBOB) or ionic liquids (ILs) are possible [62,65,66,67,68,69,70]. Electrolyte additives are used up to 5%, either by weight or by volume [71]. Due to the application of additives, the electrolyte properties can be influenced: improvement of the flammability, enabling overcharge protection [71,72,73] or the SEI formation [25,74,75,76,77,78]. The SEI is formed during the first charge/discharge (formation cycles) of the LIB cell and is essential for safety and performance due to its protection of the electrolyte from further reductive decomposition at the anode surface [79,80,81].

## 3. Aging of Lithium Ion Battery Electrolytes

For a better customer acceptance of LIBs, their capacity loss and therefore the reduction of their operating lifetime has to be minimized. The lifetime is limited by several aging mechanisms inside the cell and its components [14,15,30,82,83]. As stated in Section 2, the electrolyte is a mixture since it has to meet several requirements. Unfortunately, the salt lithium hexafluorophosphate is neither chemically nor thermally stable. A thermal equilibrium between LiPF_6_ and LiF/PF_5_ (Equation (1)) exists and PF_5_ as a strong Lewis acid then further reacts with traces of water (present due to the high hygroscopicity of PF_6_) to POF_3_ (Equation (2)). With additional water, POF_3_ is hydrolyzed to difluorophosphoric acid (Equation (3)), monofluorophosphoric acid (Equation (4)) and finally to phosphoric acid (Equation (5)) [35,84,85,86,87,88].

LiPF_6_ ⇆ LiF + PF_5_ (1)

PF_5_ + H_2_O ⇆ 2HF + POF_3_ (2)

POF_3_ + H_2_O ⇆ HF + HPO_2_F_2_ (3)

HPO_2_F_2_ + H_2_O ⇆ HF + H_2_PO_3_F (4)

H_2_PO_3_F + H_2_O ⇆ HF + H_3_PO_4_ (5)

**Figure 2:**
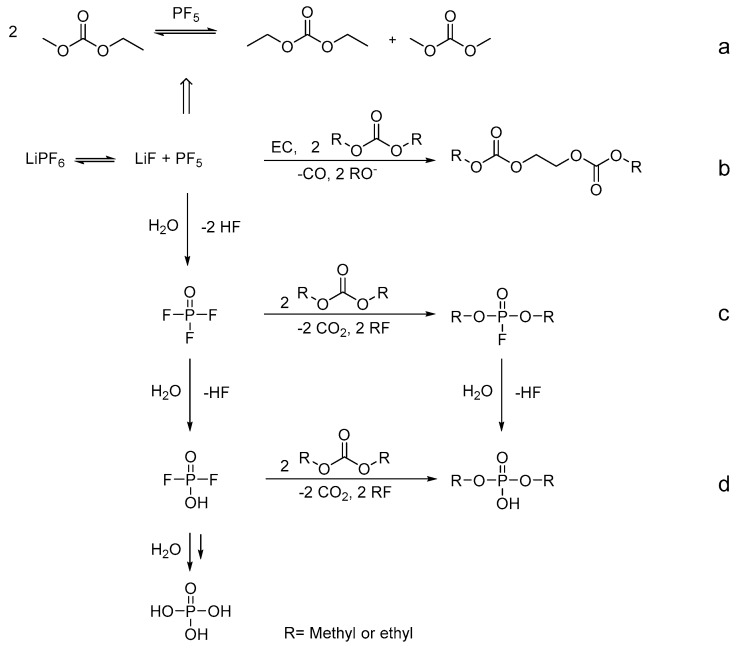
General decomposition pathways for the formation of: transesterifications products (**a**); oligocarbonate-based products (**b**); organophosphate-based products (**c**); organic fluorophosphate-based products; and hydrolysis products (**d**) [43,84,85,86,87,88,89,90,91].

PF_5_ can also initiate the transesterification of EMC to DMC and DEC (Figure 2a) [89]. Furthermore, the described hydrolysis products can react with the organic carbonate solvent to foster numerous organophosphate-based and organic fluorophosphate-based aging products (Figure 2c,d) [43]. Oligomeric carbonate-based decomposition products are formed by the electrochemically induced ring opening of EC and reaction with the linear carbonates (Figure 2b) [84,90,91]. The exact formation mechanism of the oligomeric carbonate-based compounds is still under discussion.

The formed organic fluorophosphates have a high potential toxicity, and toxicological data are available for dimethyl fluorophosphate (DMFP) and diethyl fluorophosphate (DEFP) (Table 1). The LD_50_ value represents the amount of a solid or liquid material that it takes to kill 50% of the test animals in one experiment. The LC_50_ is the corresponding value for vapors, gases or dusts [92,93,94,95,96,97,98,99,100].

The World Health Organization has five categories for hazardous substances with classified toxicity. Despite the lowered toxicity compared to sarin, according to toxicity data and classification, both DMFP and DEFP are classified in category 1, which is the most dangerous (fatal by swallowing <5 mg) and fatal by contact with the skin <40 mg/kg) [101]. Therefore, due to the P–F bond in many electrolyte decomposition products, they are considered hazardous as well. As an example, the neurotoxin diisopropyl fluorophosphate is known for its high toxicity as a consequence of the reaction with the enzyme acetylcholinesterase (AChE). The regular function of AChE is to hydrolyze acetylcholine, an essential neurotransmitter for the peripheral nervous system (PNS), which consists of the nerves and ganglia outside of the brain and spinal cord. The PNS is responsible for the connection of the central nervous system with limbs (skeletal muscle movement) and organs. By reaction between the organophosphate and AChE, a covalent bond is formed and the enzyme is stopped from regular functioning. This leads to an accumulation of unhydrolyzed acetylcholine and the symptoms of the intoxications are convulsions, suffocation and myosis, which ultimately can lead to death [102,103].

**Figure 3:**
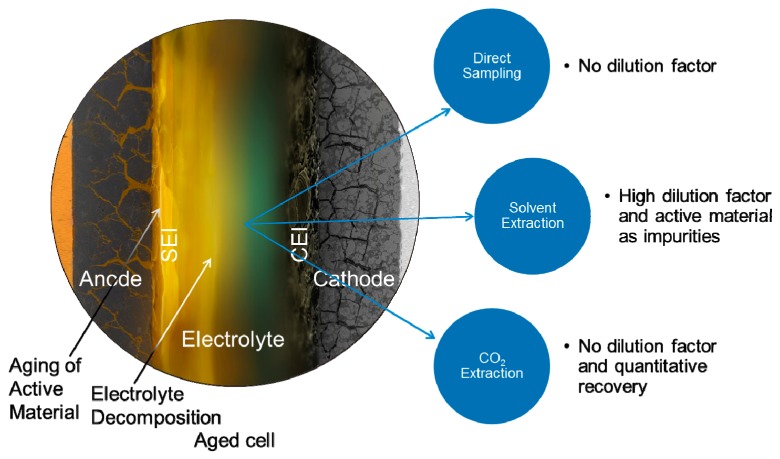
Schematic sketch of an aged lithium ion battery (LIB) and strategies for electrolyte recovery. CEI: cathode electrolyte interphase; SEI: solid electrolyte interphase.

Besides the dangers regarding working safety when handling spent lithium ion batteries or shredded recycling waste, the fluorinated compounds and hydrofluoric acid can interfere or damage industrial scaled recycling processes and therefore have to be removed before the recycling process by extraction [104]. For post-mortem analysis and aging analysis of LIB electrolytes, extraction is inevitable, as well. While the electrolyte is introduced as a liquid during cell assembly, it penetrates and immobilizes in the electrodes during electrochemical operation (Figure 3). Thus, an opened LIB appears in most of the cases as “dry” after electrochemical operation.

Direct sampling of the electrolyte from a LIB, whenever possible, is the method of choice. However, it is seldom applicable. The extraction with an appropriate solvent can yield quantitative results but significant amounts of the electrode material are extracted as well and will be present as impurities. The extraction with sub- and supercritical CO_2_ in comparison can yield quantitative results without a dilution factor.

## 4. Recycling of Lithium Ion Batteries Electrolytes

Lab-scale and commercial LIB recycling processes are focused on the recovery of the heavy metals (Ni, Co and Mn) and lithium itself from the cathode active material or on the current collectors, which consist of copper and aluminum [105,106,107]. The electrolyte is not recovered but simply combusted or disposed during the process or the handling of the electrolytes is not mentioned at all in the literature [108,109,110]. However, due to the directive 2006/66/EC from the European Parliament and the Council >50 wt % of LIB cell materials have to be recycled. The weight fraction of an electrolyte in a LIB cell is around 10–15 wt %, depending on the cell chemistry and geometry. The first approach for electrolyte recovery was the extraction with an appropriate regular solvent [108]. Nevertheless, general recycling procedures always referenced to the already published articles and procedures for electrolyte recycling [111,112,113,114]. Sloop et al. were the first to propose the application of supercritical carbon dioxide as an extraction medium for LIB electrolytes [115]. However, the given information about extraction conditions and the resulting extraction behavior is limited. In addition, the supercritical extraction procedure was not reported to be applied in a real recycling process, and not even on the lab-scale. However, compared to solvent extractions, the recycling efficiency with regard to the used amount of kg/CO_2_ per kg/shredded material is in the same region for the reported methods.

Since spent LIBs are primarily from portable consumer applications and therefore only possess a limited amount of recyclable materials, the established pyro-hydrometallurgical recycling strategy by Umicore AG & Co. KG (Brussels, Belgium) is currently the most economic process [116]. Nickel metal hydride (Ni-MH) batteries and LIBs are delivered to a furnace and molten at temperatures of up to 1450 °C. With the help of co-added slag formers, Ni, Co and Cu form an alloy, while Li, Fe, Mn and Fe accumulate inside the slag and are disposed. The organic cell components including the electrolyte are incinerated. In the following hydro-metallurgical step, the alloy fraction is separated and refined for re-utilization [116].

**Figure 4:**
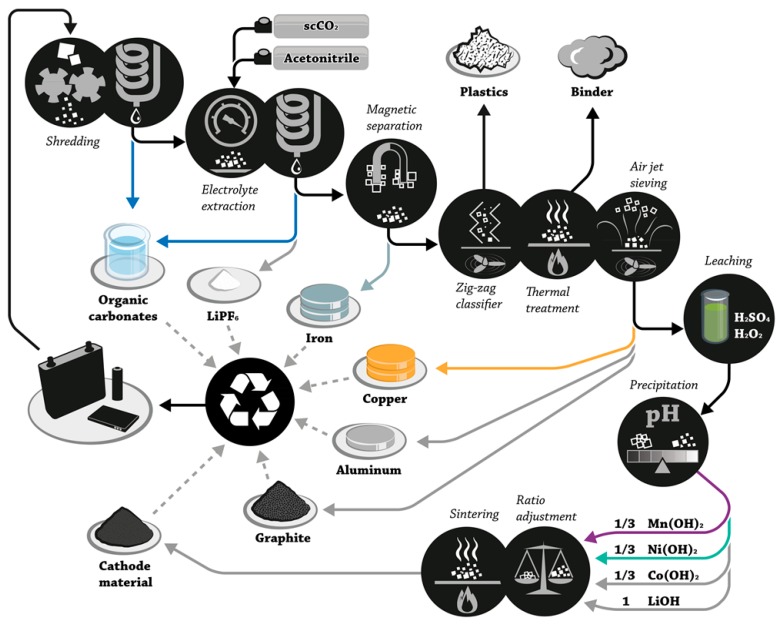
The mechanical-hydrometallurigcal “LithoRec II” recycling process. It was reproduced from reference [127], with permission from John Wiley & Sons 2016. scCO_2_: supercritical carbon dioxide.

With the growing market for xEVs, the amount of spent LIBs will drastically increase. Considering several hundred kilograms of LIB cell material per car and taking into account that similar cell chemistries are used by the manufacturers, a continuous material flow is created. Therefore, a more holistic recycling process can be applied (Figure 4), including the recovery of the electrolyte [117]. This mechanical-hydrometallurgical process aims to meet the demands of the EU directive and therefore the utilization of most cell materials from a LIB. The deep discharged (by external resistance or power) battery packs are dismantled to the individual cell level and shredded in an inert atmosphere [108,114,118]. The electrolyte evaporated during this first step is collected by condensation. The electrolyte remaining in the shredded material is subject to three possible recovery methods: (i) a thermal drying step with the disadvantage of losing the conducting salt, which is the most costly component of the electrolyte; (ii) application of supercritical CO_2_ in a static setup; and (iii) supercritical CO_2_ in a flow-through setup with co-solvents for the recovery of the whole electrolyte including the conducting salt. The latter two setups will be discussed in Section 6, here we concentrate on method (i): the shredded material is further processed by removing iron parts via a magnetic separation tool and then the electrode material is parted from the separator by air flow. The binder in the electrodes is removed by heating up to 400–600 °C which additionally causes the detachment of the current collectors from the active material particles. The graphite anode material and the copper and aluminum current collectors are taken out of the process and lithium is leached out of the cathode material, while the active material is dissolved in an acidic mixture. Several approaches are carried out to separate the heavy metals from each other: complexing agents such as di(2,4,4,trimethylpentyl)phosphinic acid [119]; or leaching with oxalate [120], ascorbic [121], citric [122] or concentrated acids [123,124,125]. However, biological approaches are also investigated [126]. The performance of the resynthesized cathode and anode material was reported by electrochemically and analytically characterized [127,128].

## 5. Carbon Dioxide and Its Extraction Properties

Besides being infamous as greenhouse gas [129,130,131,132], CO_2_ is substantial to many chemical reactions. These reactions include the application as a monomer in materials synthesis [133] or as a C1 block in the fabrication of polycarbonates [134] or other organic compounds [135]. Furthermore, CO_2_ is involved in the synthesis of linear and cyclic carbonates for LIB electrolytes as well [136,137,138,139]. It has been applied for lithium metal and lithium ion batteries as a SEI forming additive [140] and graphite anode surface modifications [141,142]. Additionally, due to the reduction or oxidation of the electrolyte CO_2_ is produced and present in LIBs [143,144].

**Figure 5:**
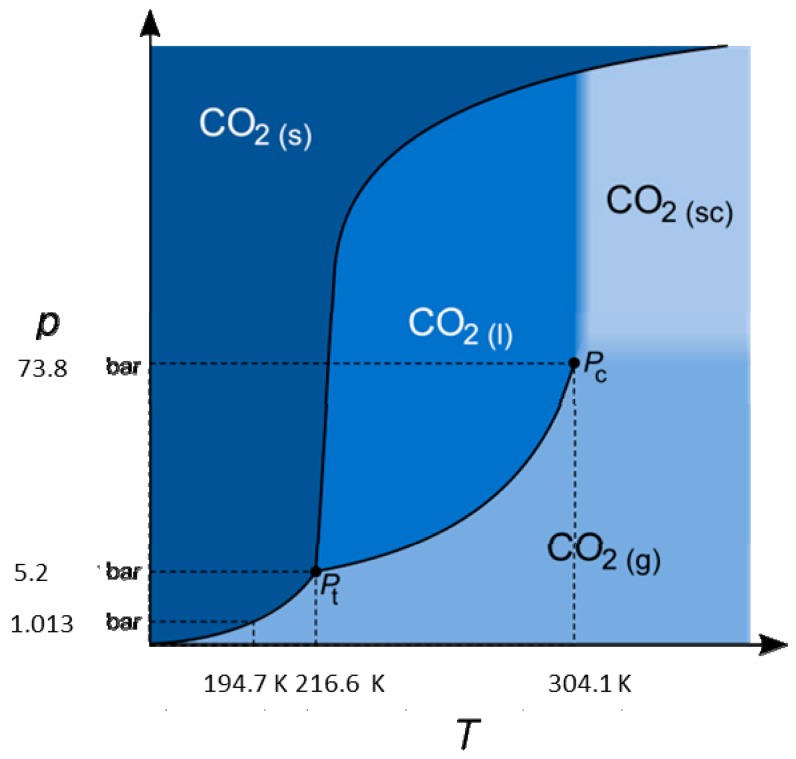
Pressure and temperature phase diagram of CO_2_ [158,159]. K: Kelvin, p: pressure, T: temperature, sc: supercritical, l: liquid, g: gaseous, s: solid.

Aside from the standard phases (solid, liquid and gaseous), the supercritical phase is reached relatively non-elaborate. Temperature and pressure need to be increased above the critical point of 31 °C and 74 bar (Figure 5) [145]. In this state, supercritical CO_2_ has the density of liquid CO_2_ and the viscosity of gaseous CO_2_. The physical properties are between those of the liquid and gaseous phase with greatly enhanced dissolution characteristics [146]. In combination with additional solvents, a wide variety of organic substances can be extracted and, therefore, supercritical carbon dioxide is the most applied supercritical medium for extraction [147]. It is normally used in food chemistry, with the well-known example of coffee decaffeination [148,149,150,151,152]. Other application areas include as a reaction medium for metal nanoparticle synthesis [153] or olefin polymerization [154], as a drying aid for electrode material synthesis [155] and crack-free silica aerogel production [156,157]. In supercritical fluid chromatography it functions as the eluent [147].

For supercritical extraction, commercial flow-through units with a corresponding compressor for the generation of supercritical CO_2_ from liquid CO_2_ are available. However, helium head pressure carbon dioxide (HHPCO_2_) can be used as an alternative. Here, liquid carbon dioxide is compressed with a helium head pressure to 120 bar. Because of the different densities, in the two-phase system, supercritical carbon dioxide is available. Nevertheless, there are small deviations in the extraction behavior of HHPCO_2_ compared to supercritical CO_2_ due to little amounts of dissolved helium in the CO_2_ phase. This results in decreasing density of the helium head pressure carbon dioxide with reduced dissolving power towards aromatic analytes [160].

Nonetheless, compared to liquid solvent extraction, subcritical and supercritical CO_2_ extraction offers unique advantages. The extraction is fast, highly selective and efficient. Furthermore, pre- or post-concentration or cleanup steps are not necessary [161].

## 6. Application of Subcritical and Supercritical CO_2_ for Recycling of LIB Electrolytes

The use of CO_2_ as an extraction medium was first mentioned in a patent by Sloop et al. Typical for a patent, the details about conditions and parameters as well as influence of the processing on the battery materials were not disclosed [115]. There are no further reports about any application of the patented process.

**Figure 6:**
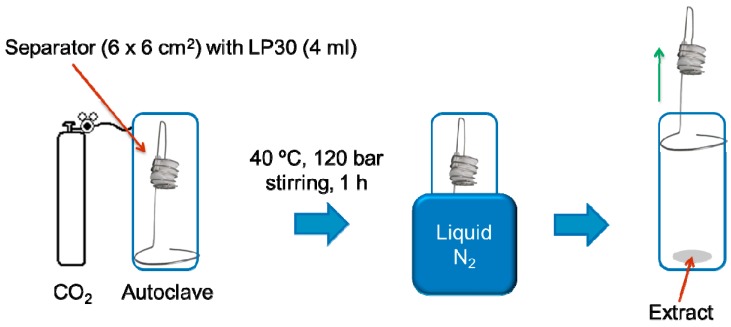
Schematic setup of the applied extraction procedure by Grützke et al. It was reproduced from reference [19] with permission from Elsevier, 2014.

The first application of supercritical CO_2_ for the extraction of LIB electrolytes was reported by Grützke et al., 2014 [19]. They applied supercritical helium head pressure carbon dioxide (scHHPCO_2_) in an autoclave and investigated the extraction behavior with a set of two different separators and electrolytes (Figure 6). The extracts were analyzed by gas chromatography–mass spectrometry and ion chromatography–electrospray ionization–mass spectrometry to determine the recovery rate and the nature of the obtained electrolyte composition. It was stated that the recovery rates and extract compositions were strongly depending on the material of which the electrolyte was extracted. The highest achieved recovery rate was 73.5 ± 3.6 wt %.

After these proof-of-principle-experiments, commercial 18,650 cells were investigated as real samples. In addition to a reference cell, which was opened and extracted after formation, i.e., as supplied, LIB cells were electrochemically aged at 20 °C and 45 °C for post-mortem studies. The extracts were again analyzed by both mentioned techniques. Beside the electrolyte constituents, the following aging products were found and characterized: dimethyl-2,5-dioxahexane dicarboxylate (DMDOHC), ethylmethyl-2,5-dioxahexane dicarboxylate (EMDOHC) and diethyl-2,5-dioxahexane dicarboxylate (DEDOHC).

In all experiments, they showed the applicability of the CO_2_ extraction. Furthermore, besides the application as a recycling tool, they showed the usefulness of the method for aging investigations on electrochemically treated cells. However, due to the obtained recovery rate, flow-through experiments with additional co-solvents were proposed in order to optimize the recovery rate.

**Figure 7:**
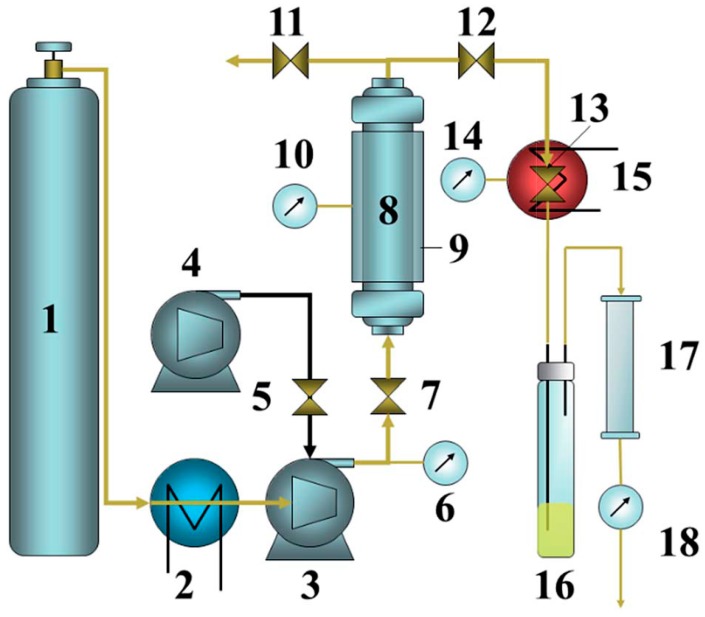
Schematic setup of the applied extraction procedure by Dai et al. with (1) CO_2_ cylinder; (2) cooling bath; (3) air driven fluid pump (gas booster pump); (4) air compressor; (5) air regulator; (6) CO_2_ pressure; (7) inlet valve; (8) extraction vessel; (9) heating jacket; (10) vessel heat; (11) vent valve; (12) outlet valve; (13) flow valve; (14) valve heat; (15) heating jacket; (16) collecting vial; (17) alumina filter; and (18) gas flow meter. It was reproduced from reference [162] with permission from the Royal Society of Chemistry, 2014.

**Figure 8:**
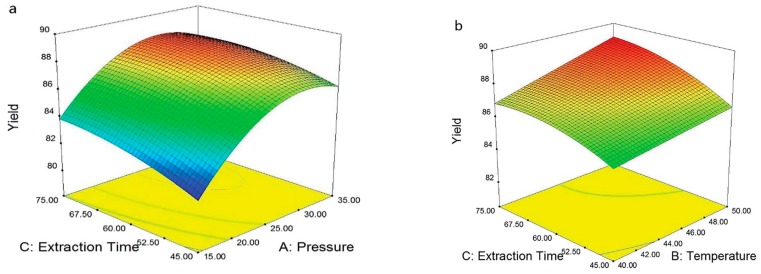
Response surfaces and contour plots for: (**a**) extraction time vs. pressure; and (**b**) extraction time vs. temperature. It was reproduced from reference [162] with permission from the Royal Society of Chemistry, 2014.

Dai et al. described an alternative approach for the recovery of LIB electrolytes from separators with a commercial extraction system [162] (Figure 7). Fourier transform infrared spectroscopy (FT-IR), gas chromatography-mass spectrometry (GC-MS), ^19^F- and ^31^P-NMR and inductively plasma-optical emission spectrometry (ICP-OES) were applied to systematically investigate the extract. The final recovery rate was determined as 85.07% ± 0.36%, however the conducting salt could not be recovered in sufficient amounts. They proposed that LIPF_6_ was hydrolyzed during the supercritical CO_2_ extraction process extraction, which was investigated with NMR to observe typical PF_6_^−^ hydrolysis products. Furthermore, the extraction parameters were optimized by varying the operating parameters (extraction pressure, temperature and static time) according to the Box–Behnken design (Figure 8). With the help of a polynomial regression model, the mildest experimental conditions were determined (23.4 MPa, 40 °C and 45 min). The predicted recovery rate, 85.22%, was in very good agreement with the experimental recovery rate of 85.07%. Additionally, the results of the experiment showed that the extraction pressure is the major decisive factor for electrolyte extraction.

In agreement with Grützke et al. [19], they emphasized the usefulness of supercritical CO_2_ as an efficient and environment-friendly electrolyte separation method.

**Figure 9:**
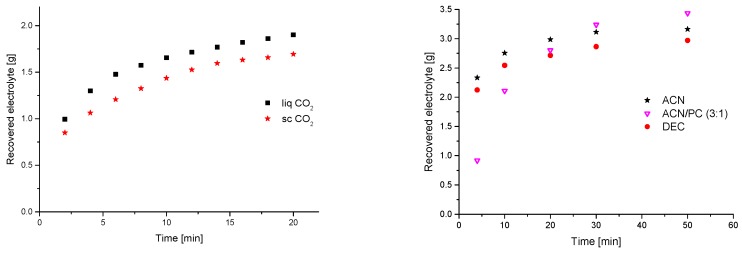
(**Left**) Time dependency of the amount of recovered electrolyte from commercial 18,650 cells after formation extracted with supercritical (300 bar, 40 °C; red stars) and liquid (60 bar, 25 °C; black squares) CO_2_; and (**Right**) time dependency of the amount of recovered electrolyte from commercial 18,650 cells after formation extracted with liquid CO_2_ and 0.5 mL/min additional solvents (black stars: acetonitrile (can); magenta triangles: ACN/propylene carbonate (PC) (3:1); red circles: diethyl carbonate (DEC)). It was reproduced from reference [163] with permission from the Royal Society of Chemistry, 2015.

**Figure 10:**
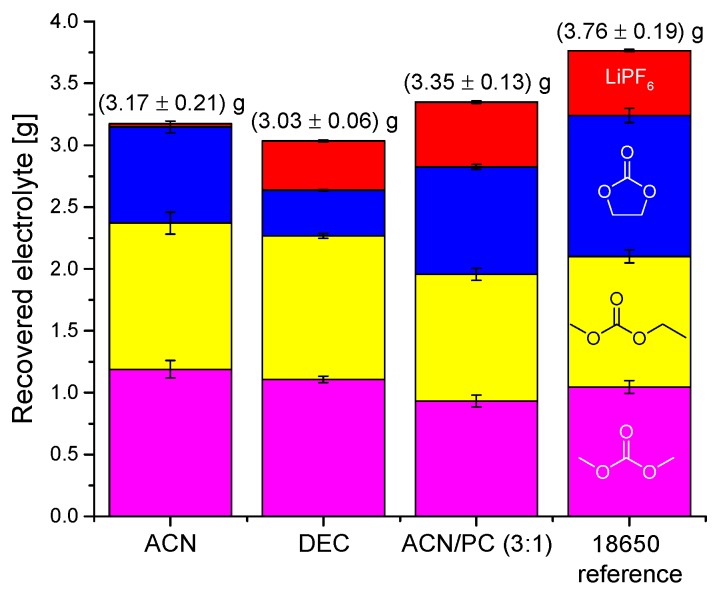
Compositions and amounts (determined with gas chromatography—mass spectrometry (GC-MS) and ion chromatography (IC)) of the recovered electrolytes from commercial 18,650 cells after formation extracted with liquid CO_2_ and additional solvents for 30 min, with subsequent 20 min without additional solvent. Red, top: LiPF_6_; blue, below: ethylene carbonate (EC); yellow, middle: ethyl methyl carbonate (EMC); magenta, bottom: dimethyl carbonate (DMC). It was reproduced from reference [163] with permission from the Royal Society of Chemistry, 2015.

Following their static experiments, Grützke et al. applied a flow-through design [163]. They used supercritical and liquid carbon dioxide (sc and liq CO_2_) under addition of different solvents for the optimized extraction of the electrolyte from commercial LiNi_0.33_Co_0.33_Mn_0.33_O_2_ (NMC))/graphite 18,650 cells (Figure 9). With 89.1 ± 3.4 wt %, the best overall recovery rate was achieved for the added ACN/PC mixture with the highest concentrations for EC and LiPF_6_ (Figure 10). In addition, they investigated the time dependency of the recovered electrolyte for both setups. It was demonstrated that the developed method was suitable for LIB electrolyte extraction and post-mortem or aging investigations of LIB cell components because a qualitative overview was achieved after a few minutes of extraction also for apparently “dry” cells, where the electrolyte is deeply incorporated and immobilized in the electrode material.

**Figure 11:**
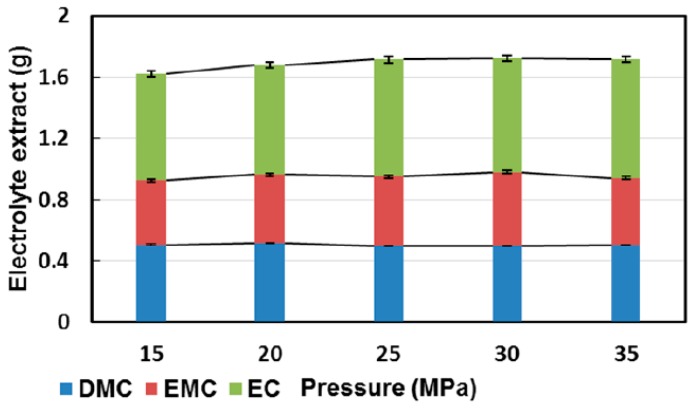
Compositions of the extracts corresponding to values obtained by gas chromatography—flame ionization detector (GC-FID) measurements. Green, top: EC; crimson, middle: EMC; blue, bottom: DMC [164].

**Figure 12:**
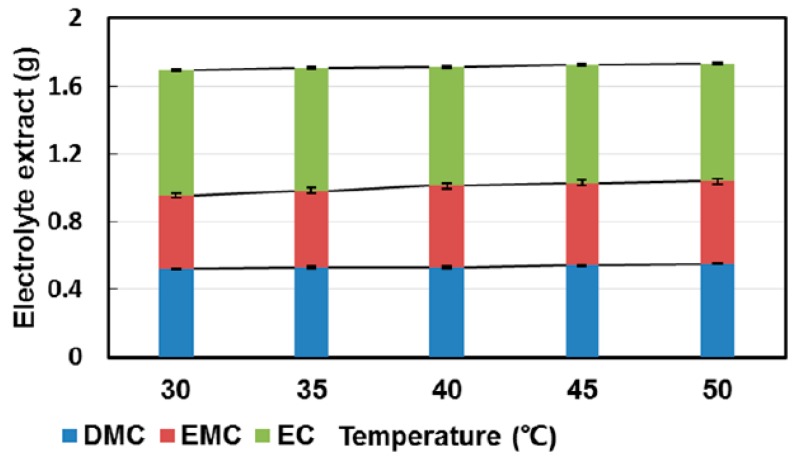
Compositions of the extracts corresponding to GC-FID measurements. Green, top: EC; crimson, middle: EMC; blue, bottom: DMC [164].

In their second work on the topic, Dai et al. applied supercritical carbon dioxide as an extraction medium for the investigation of the extraction temperature and pressure (15 to 35 MPa), temperature (30 to 50 °C) [164]. The electrolytes were again adsorbed on a separator and investigated regarding the pressure (15–35 MPa), the temperature (30 to 50 °C) and the dynamical extraction time (25 to 65 min). Afterwards, the extract was analyzed with GC-FID (flame ionization detector) (Figure 11 and Figure 12). The effect of the extraction pressure was examined using five levels from 15 to 35 MPa. The overall extraction yield was enhanced with higher pressures. This was attributed to the increased EC extraction yield under higher pressures due to the high polarity of supercritical CO_2_.

The effect of temperature was investigated in a similar way (5 levels from 30 to 50 °C). Here, the temperature showed the same trend: with raising temperature, the overall extraction yield was increased. However, the extraction of EC was contradictory to the pressure experiments. The polarity of the supercritical carbon dioxide was reduced with higher temperatures; thus, Dai et al. [164] concluded that the polarity played a more important role than the supercritical fluid density.

The overall highest recovery rate was reported as 88.71 ± 0.87 wt % but only in regard to the organic carbonate solvents. The conducting salt and aging products were not reported. As a conclusion of the extraction pressure and temperature experiments, they showed that the extraction process of carbonate is a predominantly determined by polarity. Furthermore, nonpolar carbonates should be extracted with non-polar or weakly polar extraction media. In comparison, polar carbonates should be extracted with polar solvents or with the addition of medium polarity co-solvents into non-polar solvents.

Rothermel et al. applied the methods by Grützke et al. for the extraction of electrolytes and the subsequently effect on the graphite anode recycling efficiency [127]. Therefore, they applied three different approaches for their investigations: (i) thermal evaporation of volatile electrolyte components; (ii) electrolyte extraction with subcritical CO_2_ and acetonitrile (ACN); and (iii) electrolyte extraction with supercritical CO_2_. It should be noted that they replaced the term liquid CO_2_ with subcritical CO_2_ due to the fact that the used parameters were in fact subcritical and not liquid conditions for the CO_2_. However, they concluded that the application of the supercritical carbon dioxide extraction method was unfavorable for the resulting crystallinity size of the graphite particles and therefore had an adverse impact on the electrochemical performance. In comparison, the electrolyte extraction using subcritical carbon dioxide was considered to be the “best” recycling method, as the recycled graphite showed the best electrochemical performance and the electrolyte was recovered by 90% including the conductive salt. In general, with the help of analytical and electrochemical characterization techniques it was shown that graphite originating from a previously electrochemically aged commercial cell subjected to a subcritical carbon dioxide assisted electrolyte in combination with a thermal treatment demonstrated the best electrochemical characteristics outperforming even fresh commercial synthetic graphite TIMREX^®^ SLP50 which was used as benchmark (Table 2).

## 7. Application of Subcritical and Supercritical CO_2_ as a Sample Preparation Tool

**Figure 13:**
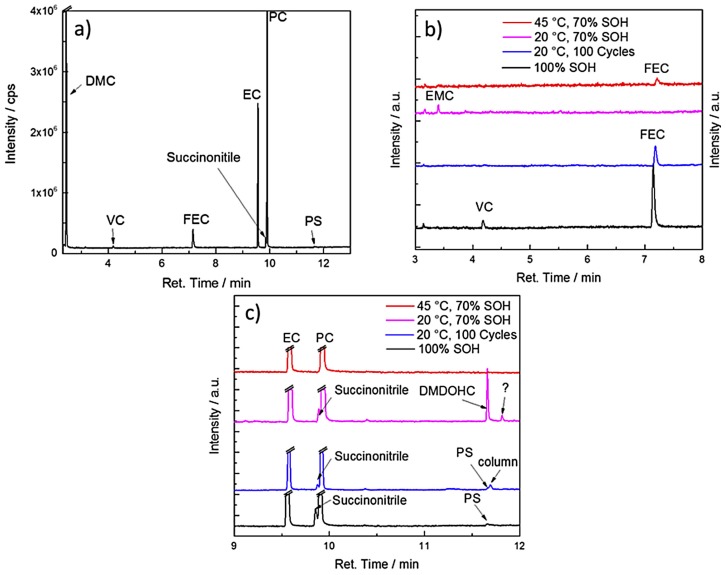
GC-MS investigations on the electrolyte of a fresh cell extracted with supercritical CO_2_ (**a**); and comparisons of fresh and aged electrolytes at significant retention times (**b**,**c**). It was reproduced from reference [165] with permission from Elsevier, 2017.

Besides minor aging investigations after supercritical or subcritical extraction (presented by Grützke et al. [19,104,163]) and the influence of the extraction method on the recyclability of graphite (reported by Rothermel et al. [127]), there is a recent study which focused primarily on aging investigations [165]. The aging experiments were conducted on commercial 18,650-type state-of-the-art cells to determine the influence of temperature during electrochemical cycling on the aging behavior of the different cell components. The cells, based on the Li(Ni_0.5_Co_0.2_Mn_0.3_)O_2_/graphite chemistry, were aged at 20 °C and 45 °C to different states of health. The electrolyte was extracted based on the methods by Grützke et al. [19,163]. With the help of electrolyte aging analysis by GC-MS, it was shown, that temperature dependent cycling leads to differences in SEI composition. The electrolyte samples which were extracted with supercritical CO_2_ from fresh and aged cells and revealed the following composition: the electrolyte of the fresh cell consisted of DMC and EC and PC as solvents, whereas fluoroethylene carbonate (FEC) [166] and succinonitrile [167] were identified as electrolyte additives in significant amounts. Furthermore, traces of vinylene carbonate (VC) [75] and 1,3-propane sultone [168] were detected in the electrolyte (Figure 13). The cell aged at 45 °C shows traces of FEC in the electrolyte after more than 1000 cycles. In contrast, the additive FEC was no longer detected in the cells cycled at 20 °C. With the help of the supercritical CO_2_ extraction, which made even traces of electrolyte additives and decomposition products available, they could conclude that after FEC consumption, the amount of organic SEI components increased and PC could co-intercalate, thus leading to a poorer performing SEI for the cells cycled at 20 °C compared to those cells at 45 °C.

## 8. Conclusions

Contrary to previous reports, the role of CO_2_ as an extraction medium is important in general and very promising for future applications as LIB cell extraction. For the sake of more refined post-mortem and aging analyses of LIB cell components, the quantitative extraction with subcritical/liquid and supercritical CO_2_ holds the advantage that there is no loss of information due to dilution factors (which occurs with regular solvents) or by missing compounds that could not be extracted due to polarity/non-polarity of the target compounds.

Since the numbers of used portable consumer products rise enormously and, more important, xEVs are penetrating the market in greater and greater quantities, new strategies are needed. Especially recycling, as growing application and research field in a holistic treatment of lithium ion batteries relies on novel, more effective and efficient process techniques such as CO_2_ extraction. Together with a strict legislation on the recycling rate and the decreasing availability of the raw battery materials, the enormous growth rates in used LIBs will become most significant for further growth in importance of this processing technique.

## Figures and Tables

**Table 1:** Toxicity data for dimethyl fluorophosphate (DMFP) and diethyl fluorophosphate (DEFP) compared to the nerve agent sarin (mus = mouse; ivn = intravenous, skn = skin; ihl = inhalation; n/a = not available).

Compound	ivn-mus (LD_50_)	skn-mus (LD_50_)	ihl-mus (LC_50_)
Sarin	0.109 mg/kg	1.08 mg/kg	5 mg/m^3^/30 min
DMFP	0.45 mg/kg	36 mg/kg	290 mg/m^3^/10 min
DEFP	n/a	35 mg/kg	100 mg/m^3^/10 min

**Table 2:** Overview of the discharge capacities and associated Coulombic efficiencies obtained from electrochemical charge/discharge cycling experiments. SubCO_2_: subcritical carbon dioxide. It was reprinted with permission from reference [127], Copyright John Wiley & Sons, 2016.

Sample (State of Health (SOH)	Coulombic Efficiency/%				Discharge Capacity 50th Cycle/mAh·g^−1^
	1st Cycle	2nd Cycle	3rd Cycle	50th Cycle	
thermal (100%)	56.1 ± 1.8	91.8 ± 0.4	94.7 ± 0.3	99.8 ± 0.1	332.7 ± 0.3
thermal (70%)	85.4 ± 0.5	97.8 ± 0.3	98.5 ± 0.3	99.9 ± 0.1	346.8 ± 7.8
subCO_2_ (100%)	81.6 ± 3.1	96.3 ± 1.2	97.6 ± 0.8	99.9 ± 0.1	372.7 ± 2.5
subCO_2_ (70%)	82.9 ± 0.9	97.6 ± 0.1	98.5 ± 0.1	99.9 ± 0.1	379.9 ± 4.4
scCO_2_ (100%)	78.7 ± 1.2	96.2 ± 0.4	97.4 ± 0.4	99.9 ± 0.1	348.8 ± 1.9
scCO_2_ (70%)	82.0 ± 1.4	97.2 ± 0.3	98.2 ± 0.2	99.9 ± 0.1	375.0 ± 1.0
benchmark	84.8 ± 0.8	97.3 ± 0.2	98.2 ± 0.2	99.9 ± 0.1	357.6 ± 1.4
